# Automatic risk prediction of intracranial aneurysm on CTA image with convolutional neural networks and radiomics analysis

**DOI:** 10.3389/fneur.2023.1126949

**Published:** 2023-06-29

**Authors:** Yuan Xie, Shuyu Liu, Hen Lin, Min Wu, Feng Shi, Feng Pan, Lichi Zhang, Bin Song

**Affiliations:** ^1^School of Biomedical Engineering, Shanghai Jiao Tong University, Shanghai, China; ^2^Department of Radiology, West China Hospital of Sichuan University, Chengdu, China; ^3^Department of Research and Development, Shanghai United Imaging Intelligence Co., Ltd., Shanghai, China; ^4^Department of Pulmonary Medicine, Shanghai Chest Hospital, Shanghai Jiao Tong University, Shanghai, China

**Keywords:** intracranial aneurysm, risk estimation, feature extraction, classification, machine learning

## Abstract

**Background:**

Intracranial aneurysm (IA) is a nodular protrusion of the arterial wall caused by the localized abnormal enlargement of the lumen of a brain artery, which is the primary cause of subarachnoid hemorrhage. Accurate rupture risk prediction can effectively aid treatment planning, but conventional rupture risk estimation based on clinical information is subjective and time-consuming.

**Methods:**

We propose a novel classification method based on the CTA images for differentiating aneurysms that are prone to rupture. The main contribution of this study is that the learning-based method proposed in this study leverages deep learning and radiomics features and integrates clinical information for a more accurate prediction of the risk of rupture. Specifically, we first extracted the provided aneurysm regions from the CTA images as 3D patches with the lesions located at their centers. Then, we employed an encoder using a 3D convolutional neural network (CNN) to extract complex latent features automatically. These features were then combined with radiomics features and clinical information. We further applied the LASSO regression method to find optimal features that are highly relevant to the rupture risk information, which is fed into a support vector machine (SVM) for final rupture risk prediction.

**Results:**

The experimental results demonstrate that our classification method can achieve accuracy and AUC scores of 89.78% and 89.09%, respectively, outperforming all the alternative methods.

**Discussion:**

Our study indicates that the incorporation of CNN and radiomics analysis can improve the prediction performance, and the selected optimal feature set can provide essential biomarkers for the determination of rupture risk, which is also of great clinical importance for individualized treatment planning and patient care of IA.

## 1. Introduction

Intracranial aneurysm (IA) is a localized weak or thin spot on a brain artery, which generally balloons or bulges out and is filled with blood. Intracranial aneurysms (IAs) are commonly believed to result from a combination of genetic and environmental factors. Congenital defects in the arterial wall, including thinning or weakening of the vessel walls, can increase the risk of an aneurysm forming (1–3). The bulging aneurysm presses on brain nerves or tissues, which may burst or rupture and lead to hemorrhage. The ruptured aneurysm can cause serious health problems such as hemorrhagic stroke, brain damage, coma, and even death (4). For example, subarachnoid hemorrhage (SAH) caused by a ruptured aneurysm is life-threatening with a fatality rate of above 40% and can cause life-long cognitive impairment (5).

Current medical imaging methods for cerebral IA diagnosis include digital subtraction angiography (DSA), magnetic resonance angiography (MRA), and computed tomography angiography (CTA). Although DSA is still considered the gold standard for IA diagnosis, CTA has been proven to be an efficient method with lower cost and easier access for most patients in the actual clinical scenario (6, 7). 3D-CTA can provide detailed visualization of the anatomical structures of blood vessels in the brain and can characterize the relationship between the aneurysm and its surrounding spatial structure more comprehensively.

Although doctors can detect intracranial aneurysms based on the CTA images, it remains challenging for predicting if they are prone to rupture. It may cause difficulties in choosing preventive or conservative treatments as the former may face high surgical risk while the latter has the risk of cerebral hemorrhage caused by ruptured aneurysms. Therefore, an accurate aneurysm rupture risk prediction method is highly in demand for the treatment planning and patient care of aneurysms.

Several statistical studies have investigated risk factors for the rupture of IA, which include the aneurysm's morphology, hemodynamics, and patient-specific factors (8–13). Furthermore, Greving et al. (14) conducted a systematic review and pooled individual data analysis from 8,382 participants with subarachnoid hemorrhage as the outcome. The practical risk score assessment named PHASES was developed based on their findings. It has become one of the major assessment methods for predicting the 5-year rupture risk of unruptured IAs. In addition, some common biomechanical and hemodynamic methods have also been used for IA rupture risk estimation. Meng et al. (15) proposed an image-based computational fluid dynamic model, which demonstrated the association between hemodynamics and the rupture risk of IA.

Recently, many attempts have also been made to construct the IA rupture risk prediction models using machine learning (ML) technologies such as K-nearest neighbors (KNN) (16, 17), random forest (RF) (18, 19), support vector machine (SVM) (20), and neural networks (21). For example, An et al. (16) used five distinct classification models (XGBoost, KNN, RF, SVM, and LR) for IA rupture risk prediction with multi-dimensionally fused features. Zhu et al. (22) also adopted multiple ML methods (SVM, RF, and ANN) for IA stability assessments based on clinical features and morphological features from 3D DSA. Shi et al. (23) integrated clinical, morphologic, and hemodynamic features to build a composite model and compared the performance between several ML models (SVM, RF, LR, and multilayer perceptron) on the rupture risk prediction task of small aneurysms using CTA. To enhance the assessment of lesion characteristics in medical imaging, radiomics has been introduced to offer more comprehensive features such as shape and texture. The extracted radiomics features are then fed into machine learning algorithms for analysis. For example, Alwalid et al. (24) conducted a radiomics analysis on CTA images of patients with ruptured aneurysms and selected the most important features to construct a logistic regression model.

Recently, there have been significant improvements in medical image processing using deep learning technology. Deep learning methods such as convolutional neural network (CNN) can learn complex features from medical images and construct models with advantageous performance. Several studies have demonstrated the effectiveness of deep learning for diagnosing and predicting the progression of brain diseases (25–28). For instance, Jnawali et al. (29) proposed a fully automated deep learning framework that learns to classify brain hemorrhage cases based on cross-sectional CT images. Dai et al. (30) applied deep learning to facilitate the detection of cerebrovascular aneurysms on CTA scans. Bizjak et al. (31) proposed a deep-shaped feature extraction model that uses PointNet++ architecture to predict the growth and rupture risk of the aneurysm using CTA and MRA images. Li et al. (32) proposed a deep learning method that can directly apply to 3D CTA data without the need for manually measured features. Turhon et al. (33) proposed a deep learning model based on multi-omics factors. These studies indicate that deep learning methods can effectively extract key features from medical images for the diagnosis of brain-related diseases. However, it is essential to note that deep learning methods require a substantial amount of training data to create an effective encoder for feature extraction. As collecting a large number of medical image samples is often expensive and challenging, it is also crucial to develop robust classification methods that can make use of comprehensive features without the need for a large quantity of training data.

In this study, we proposed a novel framework for estimating the risk of cerebral aneurysm rupture. To achieve this, we proposed to extract features from CNN, radiomics, and clinical information. In turn, we applied a feature selection method to obtain an optimal feature set that is highly correlated with the patient's IA rupture information. Finally, we employed SVM to perform the final classification. The proposed method utilizes complex feature extraction techniques such as deep learning, radiomics, and machine learning to extract intricate features from IA images and clinical information. Our model offers better adaptability for classification in situations with limited datasets and realizes effective feature fusion that combines radiomics information, CNN information, and machine learning to improve the performance of aneurysm rupture risk prediction.

The main contributions of this study can be summarized as follows:

(1) We proposed a framework that integrates deep learning, radiomics, and clinical features to estimate the rupture risk of intracranial aneurysms from a more comprehensive perspective.
(2) We proposed a method that combines deep learning techniques with machine learning to achieve better classification performance.

## 2. Materials and methods

### 2.1. Materials

There were two datasets used for model construction and validation in our study.

The 301 dataset was collected from the Cooperative Beijing 301 Hospital. The 301 dataset has 239 CTA images with their corresponding segmentation of the aneurysm. After data cleaning, a total of 106 IA cases were included in the analysis due to incomplete patient information in some CTA images. The ground truth of UIA/RIA was based on the follow-up of the patients' statuses, and IAs were manually segmented by the clinical experts. Note that informed consent was obtained from all patients for the use of their information including clinical records and CTA images.

The Large IA Segmentation Dataset (LIASD) (https://doi.org/10.5281/zenodo.6801398) (28) is an open-source dataset containing 1338 CTA images with the corresponding segmentation and follow-up information.

The clinical information for the two datasets mainly includes gender, age, and the risk status of the aneurysm. Detailed demographic patient information can be found in Table 1. Each aneurysm can be either unruptured IA (UIA) or ruptured IA (RIA).

**Table 1:** Demographic and clinical information of all samples in the two datasets.

**Category**	**301 dataset**	**LIASD dataset**
Age (years): Mean **±** Std	57.3**±**12.3	57.7 ± 12.9
Gender: male/female (%)	28/78 (35.9)	571/767 (74.4)
UIA/RIA^*^	78/28	822/516

^*^UIA and RIA stand for unruptured and ruptured IA, respectively.

**Figure 1:**
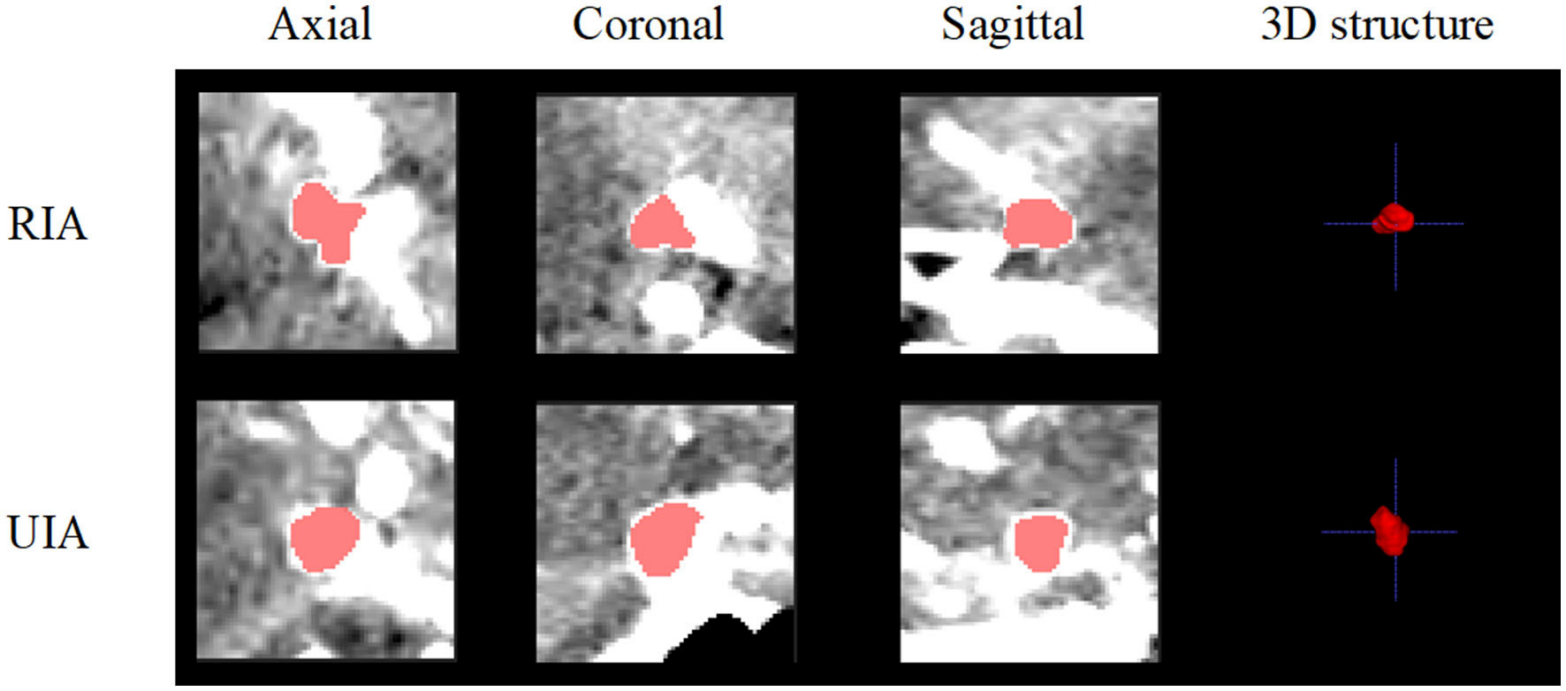
Examples of rupture and unruptured aneurysms.

**Figure 2:**
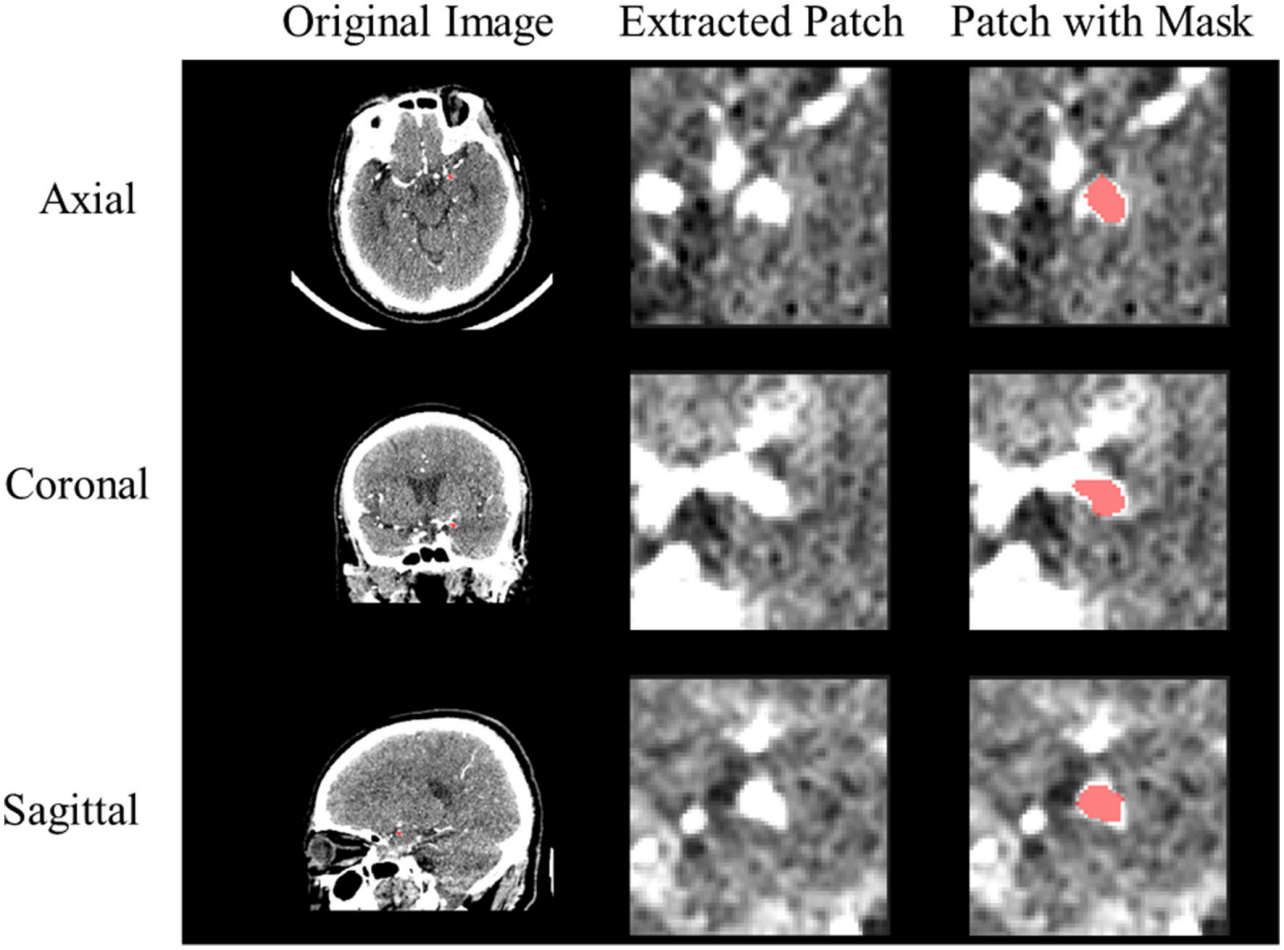
Example of preprocessed CTA image. The red color represents the IA segmentation mask from the extracted 3D patch.

Figure 1 shows examples of rupture and unruptured aneurysms, and it is difficult to distinguish if they are UIA or RIA directly from the image. In this way, we pre-process the images to make them more convenient to use. Since the raw CTA images have different voxel spacing, we rescaled all of them to the same physical size. Specifically, each voxel in the image should correspond to its appropriate physical size, by rescaling all CTA images to 0.39 × 0.39 × 0.39*mm*^3^. Based on the provided aneurysm segmentation annotations, we extracted the bounding box of each aneurysm in all CTA images. We extracted a patch for each aneurysm by setting the center of the patch as the center of the corresponding bounding box. We extracted each patch as a 3D cube of 64 × 64 × 64 in voxel space. This method ensures that the extracted 3D patch contains sufficient information on the vascular structure while avoiding the degradation of the performance caused by the extract's excessive size. Examples of the CTA images with their extracted patches are shown in Figure 2. We also normalized the image intensity by setting CT window width (WW) *to* 110*Hu* and window level (WL) *to* 40*Hu* based on clinical experience.

### 2.2. Methodology

**Figure 3:**
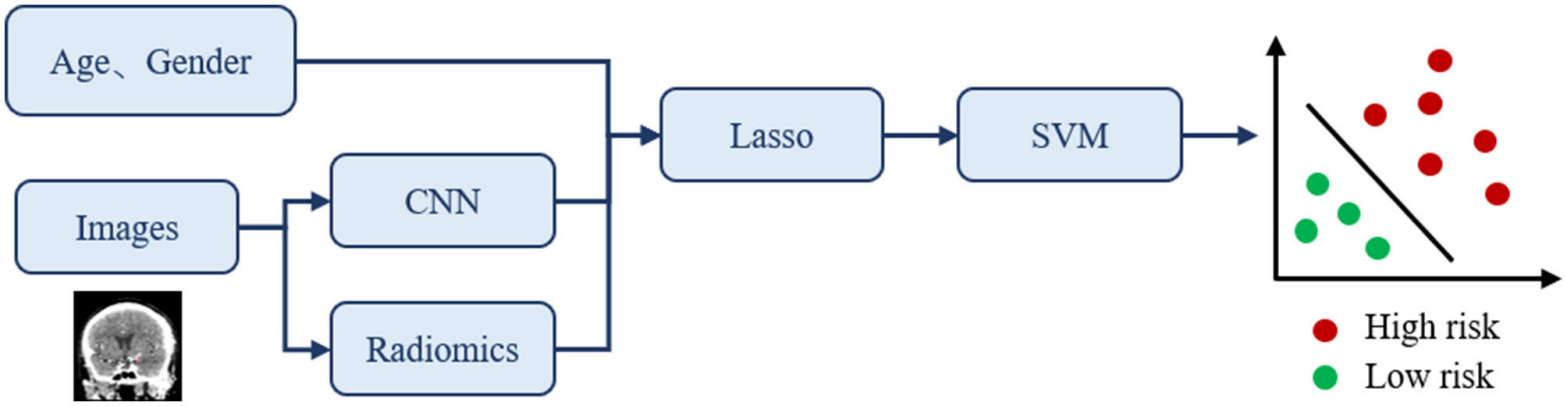
The proposed classification framework for IA rupture risk estimation. The input of this classification network is clinical information and CTA images, and the output is the prediction of risk status.

To predict the risk status of IA, we proposed a novel classification framework that combines CNN and radiomics technology as shown in Figure 3. Note that CNN is used to quantitatively describe the highly sophisticated image features, while radiomics is used to quantitatively describe the traditional image features. Therefore, the main idea is to obtain both the radiomics and CNN visual features from the collected images with the annotated region and incorporate the patient's clinical information for constructing the overall feature vector. Then, we used the LASSO regression method to find the optimal subset of features that are highly correlated with the prediction outcome. This can eliminate redundant information and simplify the model for preventing overfitting issues. Finally, the selected features were used to train the required classifier through SVM.

#### 2.2.1. CNN feature extraction

Deep convolutional neural networks have the ability to extract deep features from images. Our 3D CNN architecture has been developed from the ResNet network (34), which is a classical deep convolutional neural network for analyzing images. Since ResNet has different configurations according to their layer number settings, we use ResNet-18 as the backbone to extract CNN features, which is sufficient to extract image features. As the input data were three-dimensional, and ResNet was originally designed for two-dimensional images, we replaced the 2D convolutional layer and 2D pooling layer of ResNet-18 with a 3D convolutional layer and 3D pooling layer. The input of ResNet-18 is the preprocessed 3D patch with the aneurysm lesion, and the output is the predicted rupture risk. In the process of feature fusion, we used the trained model to extract CNN features by extracting the deep feature vectors before fully connected layers. Note that we also tried VGG as the backbone in the experiments, which is also widely applied for extracting deep learning features. However, its performance was not comparable with that of ResNet. In addition, we used random flipping for data augmentation to guarantee the robustness of the trained model.

#### 2.2.2. Radiomics feature extraction

We used PyRadiomics (35) to extract radiomics features from the 3D patch and the segmentation map. PyRadiomics is an open-source Python package for medical image processing, analysis, and interpretation. These features can be sub-divided into seven classes: First Order Statistics, Shape (3D), Gray Level Co-occurrence Matrix (GLCM), Gray Level Run Length Matrix (GLRLM), Gray Level Size Zone Matrix (GLSZM), Neighboring Gray Tone Difference Matrix (NGTDM), and Gray Level Dependence Matrix (GLDM). We hypothesize that these features can provide helpful additional information for predicting the risk of rupture since these contain relatively deep morphology and texture features of the aneurysm.

#### 2.2.3. LASSO feature selection

After feature extraction, we had a total of 650 features, including 512 CNN features, 136 radiomics features, and 2 clinical features (age and gender). Among these features, some may not be relevant to rupture risk prediction. Therefore, we used the least absolute shrinkage and selection (LASSO) regression to select features that have a strong correlation with rupture risk and to prevent the issue of overfitting while constructing the classifier. The LASSO regression is a model in which the L1 norm constraint term is added to the cost function of the linear regression model. The optimization goal can be represented as Equation (2.1). It conducts variable screening and complexity adjustment through the penalty coefficient λ:


(2.1) $$\begin{array}{l} \begin{array}{l} \underset{w}{min}=\sum_{i=1}^{m}{\left(y_{i}-w^{T}x_{i}\right)}^{2}+\text{λ}||w|{|}_{1}. \end{array} \end{array}$$

#### 2.2.4. SVM-based risk status prediction

In this study, we used SVM to predict the risk of aneurysm rupture after feature extraction and selection. SVM is one of the most popular supervised learning algorithms in classification and regression problems. The algorithm is lightweight and efficient and has an excellent performance in high-dimensional vector space, which is more suitable in the scenario where the dataset has limited image samples. Note that in experiments, we also compared its performance with the fully connected layers of the ResNet. Each IA image has a selected feature vector with its ground truth of UIA/RIA labels, which is used to train the classifier via SVM.

## 3. Experiments and results

Since the 301 dataset is relatively small (106 cases total) for training an aneurysm rupture risk prediction task, the model is easy to be overfitted during the training process and hard to obtain acceptable performance (accuracy = 70.83% on the 301 test set). Therefore, we first used the large dataset, which is the LIASD dataset to train our baseline model (ResNet-18) and obtained the pre-trained model. Based on the pre-trained model, we used the 3-fold cross-validation to finetune and evaluate our proposed model. Deploying pre-trained models designed for larger datasets on smaller counterparts is beneficial as it provides a plausible solution to the issue of limited sample size. Moreover, we conducted an ablation analysis to ascertain the significance of the techniques in our framework toward the enhancement of rupture risk prediction.

Specifically, we have three types of features during the experiment: features extracted from the CTA image patch using ResNet-18 (Deep Features); radiomics features extracted from the original CTA image and the corresponding aneurysm masks using PyRadiomics (Radiomics Features); and clinical information of patients includes gender and age (clinical features). To evaluate the improvement of our approach, we conducted our cross-validation experience using the following five methods with different configurations:

(1) ResNet: Fine-tune the pre-trained ResNet-18 with only the CTA images to obtain the final classification.
(2) ResNet + SVM: Feed deep features to the SVM classifier to generate the final classification results.
(3) FCB-ResNet (feature concatenate before FC layer): Concatenate deep features, radiomics features, and clinical features before fully connected layers of ResNet-18, and then obtain the prediction result of ruptured IA.
(4) FC-SVM (feature concatenate + SVM): First, feed the concatenate deep features, radiomics features, and clinical features, and then feed the fusion feature vector to the SVM classifier to generate the final classification results.
(5) FC-LSVM (feature concatenate + LASSO + SVM, the proposed method): Feed the concatenate deep features, radiomics features, and clinical features. Then, select features of high importance using LASSO regression, and finally, feed the fusion feature vector to the SVM classifier to generate the final classification results.

The experimental platform of our study is the Debian 5.16.12 operating system. We performed all experiments on an NVIDIA 3090 Ti GPU.

The F2 score is the weighted harmonic mean of the precision and recall, which gives more weight to recall than to precision. For the task of predicting rupture risk, false-negatives are considered worse than false-positives. Therefore, the F2-score is also considered the main evaluation metric besides accuracy in this study.

**Figure 4:**
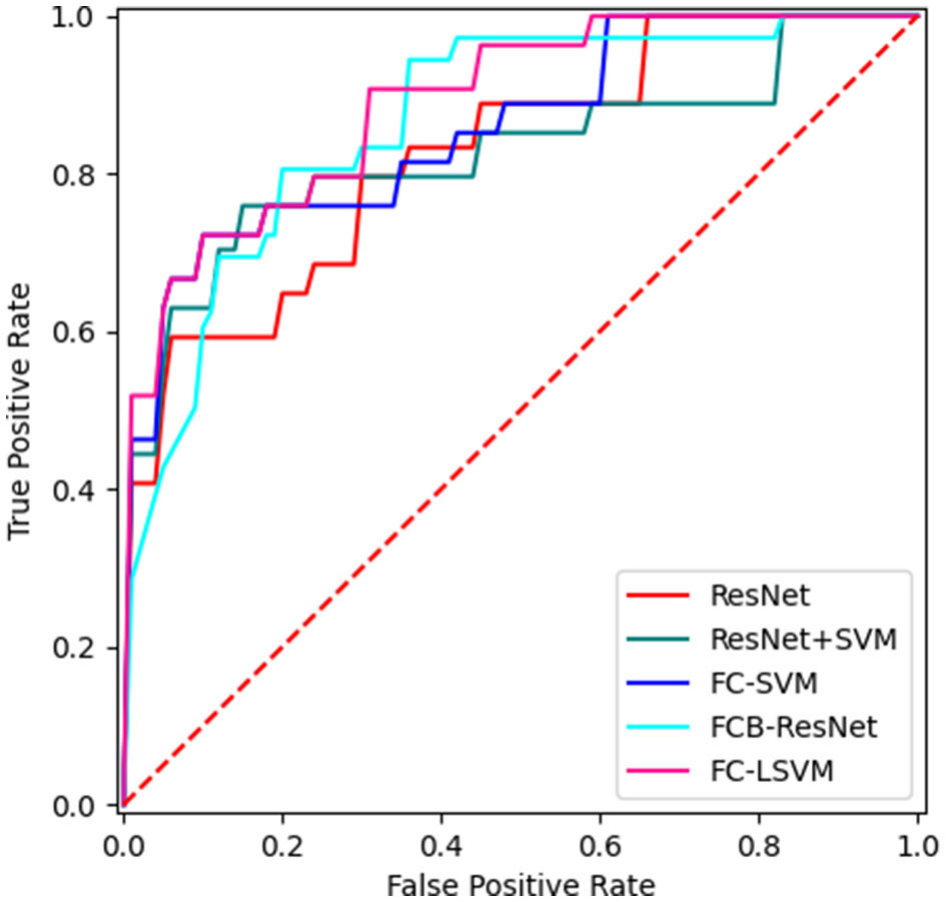
The ROC curves of the 5 alternative methods.

The experimental results are shown in Table 2, in which ResNet-18 is our baseline method as previously mentioned. It can be observed from Table 2 that the SVM classifier performs better than the original fully connected layers in the ResNet-18 model, with an improvement of 9.40% in accuracy compared with the baseline model. As envisioned earlier, the clinical information of patients and the radiomics features have offered more information for the rupture risk evaluation task since the accuracy rises by 6.84% compared with the baseline model just by adding these features before the fully connected layer. However, when operating the two lifting facts at the same time, we did not observe a further increase in accuracy, and the recall rate decreased significantly by over 10%. In addition, although SVM is good at handling high-dimensional information, its performance is highly dependent on the quality of the feature vectors, which means too much redundant information may instead reduce the performance. Table 2 shows that the proposed method with feature selection obtained the best performance with an accuracy of 89.78 ± 4.79% and an F2-score of 79.06 ± 5.70%. The area under the curve (AUC) of the receiver operating characteristic (ROC) curve can measure the quality of the classification model, and a higher AUC represents better performance. The corresponding ROC curve is presented in Figure 4.

**Table 2:** The 3-fold cross-validation results of the five alternative methods.

	**Accuracy**	**Recall**	**Precision**	**F2-score**	**AUC**
ResNet	0.7778	0.8222	0.7302	0.7006	0.8291
ResNet+SVM	0.8718	0.6297	0.8333	0.6587	0.8301
FCB-ResNet	0.8462	0.7778	0.5556	0.5727	0.8667
FC-SVM	0.8590	0.5926	0.7889	0.6047	0.8576
**FC-LSVM**	0.8978	0.7963	0.8139	0.7906	0.8909

Note that by using LASSO regression, we eliminated numerous redundant features and discovered that 61 features are highly correlated with the risk of aneurysm rupture. The 61 features can be grouped into three categories: clinical features, radiomics features, and CNN features. Table 3 provides specific measurements of the dimensions of the three groups of features. We elaborate on both the name and the characteristics of each clinical and imaging feature to aid in interpreting the features. Note that we do not provide further information for the selected CNN features since they are extracted from the constructed model and their feature representations are generally impractical to explore.

**Table 3:** Overview of features after the LASSO regression feature selection process.

**Feature group**	**Feature name**	**Description**
Clinical features (1-dimensional)	Gender	The gender of the patient
Radiomics features (9-dimensional)	Diagnostics Mask Original Volume Num	The number of aneurysms of a patient (image)
	Original Shape Maximum 3D Diameter	The maximum 3D diameter of the aneurysm
	Original Shape Sphericity	The measure of the roundness of the shape of the aneurysm region relative to a sphere.
	Original Shape Surface Area	The surface area of the aneurysm
	Original GLCM MCC	The maximal correlation coefficient (MCC), a measure of the complexity of the texture
	Original GLSZM Small Area Low Gray Level Emphasis	Small Area Low Gray Level Emphasis (SALGLE) measures the proportion in the image of the joint distribution of smaller size zones with lower gray-level values
	Original GLSZM Zone Entropy	Zone entropy (ZE) measures the uncertainty/randomness in the distribution of zone sizes and gray levels. A higher value indicates more heterogeneity in the texture patterns.
	Original GLSZM Zone Percentage	Zone percentage (ZP) measures the coarseness of the texture by taking the ratio of the number of zones and number of voxels in the ROI
	Diagnostics Mask Original Bounding Box	The location of the aneurysm in the brain
CNN features (51-dimensional)	Features extracted by ResNet-18	Part of the features in the feature map extracted by ResNet-18

We then used the Delong test to observe the significance of different methods, and the *P*-values are listed in Table 4. Although our model performs best in terms of performance, there is no statistical difference between the different models. We consider that the high *p*-value is caused by the small size of the dataset and the imbalance of sample numbers.

**Table 4:**
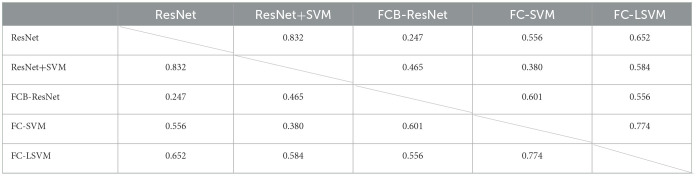
*P*-values of Delong's test.

We also compared our method with the alternative study by Liu et al. (3) and Li et al. (32). Liu et al. extracted morphological features manually and combined them with data distribution features extracted using PyRadiomics and CNN network and tried both XGBoost and FCN for final classification. Li et al. proposed a deep learning method called TransIAR net that can be directly applied to 3D computed tomography angiography (CTA) data without manually measured features. The method used a multiscale 3D CNN and a transformer encoder to extract the structural patterns and spatial dependence of the aneurysm and its neighborhood. The comparison results are shown in Table 5, in which our method improves accuracy by 3.3% (compared to 0.865), recall by 9.6% (compared to 0.700), and the F2-score by 6.2% (compared to 0.729). However, the method proposed by Liu et al. showed better precision (0.875). Overall, we still consider that our method outperformed the work of Liu et al. as accuracy and the F2-score are more important in the rupture prediction scenario.

**Table 5:** Comparison with other rupture risk prediction methods.

	**Accuracy**	**Recall**	**Precision**	**F2-score**
XGBoost	0.652	0.700	0.583	0.673
FCN	0.826	0.700	0.875	0.729
TransIAR	0.865	0.667	0.740	0.670
FC-LSVM	0.898	0.796	0.814	0.791

## 4. Discussion

In summary, we proposed a novel feature fusion framework for aneurysm rupture risk prediction. Our approach combines the features extracted by CNN with the radiomics features and clinical information of patients, filters the features using LASSO regression to provide high-quality input to the SVM classifier, and finally achieves high accuracy (0.8978 ± 0.0479) and F2-score (0.7906 ± 0.0570). The importance of the selected features in assisting the diagnosis of aneurysms is later discussed in this section.

We successfully addressed the problem of overfitting during model training and poor generalizability due to the limited size (106 cases totally) and uneven distribution of the 301 dataset using a pre-training approach on the larger LIASD dataset, followed by fine-tuning on the 301 dataset. By using this strategy, the classification accuracy of our model on the 301 dataset improved by 18.95% (89.78% vs. 70.83%). We further analyzed the selected features in experiments and summarized the advantages and disadvantages of our approach. As previously mentioned, we finally obtained the 61-dimensional feature vector for each aneurysm to predict the rupture risk, which is considered to have high correlations with the rupture risk. The optimal 61-dimensional feature vector contains three types of vectors:

(1) 1-dimensional vector concerning the clinical information of the patient
(2) 9-dimensional vector concerning the radiomics features
(3) 51-dimensional vector extracted by ResNet.

We focused on age and gender as clinical factors since previous studies have indicated their association with the rupture risk of intracranial aneurysms. For the two clinical features, gender is finally selected, indicating that there is a high correlation between gender and rupture risk. In our dataset, more patients are female, and women had a lower risk of aneurysm rupture than men. One study has shown that UIAs are more common in women than men (36). Differences between genders in the incidence of SAH have been consistently concerned since SAH disproportionally affects women. A prospective study of SAH in Texas between 2000 and 2006 showed that women have an age-adjusted risk ratio of 1.74 compared to men (37). It should be noted that, despite previous research suggesting that the risk of IA increases with age (38), the feature on age was excluded during feature selection. We attribute it to the fact that the age range of the two datasets is concentrated between 50 and 65 (the patients' age in the LIASD and 301 datasets is 57.7 ± 12.9 and 57.3 ± 12.3), which undermines its influences on the rupture risk prediction work.

Morphological features selected from the radiomics feature group for analysis primarily describe the morphological characteristics of aneurysms, including their shape, size, and surface area. These features are relatively easy to interpret and are essential for accurate diagnosis and treatment planning. It is generally believed that aneurysm size is the most significant factor affecting the risk of aneurysm rupture. It is widely recognized that the likelihood of aneurysm rupture has a linear relationship with the diameter of the aneurysm (39). The shape, size, and surface area of the aneurysm may combine to reflect the pressure of blood on the aneurysm wall, suggesting hemodynamic characteristics near the aneurysm. Studies have also shown that systolic blood pressure (SBP) is a strong predictor of aneurysm rupture (40, 41). These characteristics reflect the possibility of aneurysm rupture from the aspect of biomechanical factors.

In addition to morphological features, features that describe the gray-level information of the original CTA image were also selected. These features describe the contribution and co-occurrence of gray levels, providing valuable insights into the context and location information of the aneurysm. We believe that the heterogeneity and coarseness of the texture could indicate the malignancy of an aneurysm. According to a multivariate analysis published by Lacent, aneurysm location is a predictor of brain hemorrhage. The most frequent site of aneurysm rupture is the tip of the basilar artery, followed by the cavernous artery and posterior communicating artery as the reference group (42). In conclusion, the abovementioned features selected from the clinical feature group and the radiomics feature group were consistent with clinical experience and prior explorations.

CNN features were selected from the feature map generated by ResNet-18. As a classical deep convolution network, ResNet-18 can extract more comprehensive features that can characterize the properties of the target lesions. Furthermore, the deep features extracted by CNN from images, the radiomics information describing the morphological and texture features of the aneurysm and its contextual environment, and the patient's personal information such as gender are complementary to each other in the aneurysm rupture risk prediction task.

As a limitation of our study, it should be noted that the clinical information of patients in the two datasets only contains the age and gender, and more information can be collected in the future to further explore if they can contribute to the improvement of the rupture risk prediction task. Additionally, although the current feature fusion method is proved effective via experiments, it remains simple and more investigations can be made for designing the feature fusion strategy to further improve the performance of our approach.

## 5. Conclusion

In this study, we propose a novel classification framework to predict the risk status of IA. Specifically, image features are extracted using both CNN and radiomics and combined with patients' clinical information for predictions. Our proposed framework outperforms all other methods, with the highest measures of accuracy, F2-score, and AUC of ROC. In future work, we will investigate the use of domain adaptation techniques to enhance the robustness and accuracy of our proposed method for application in multi-site scenarios.

## Data availability statement

The original contributions presented in the study are included in the article/supplementary material, further inquiries can be directed to the corresponding authors.

## Ethics statement

Written informed consent was obtained from the individual(s) for the publication of any potentially identifiable images or data included in this article.

## Author contributions

LZ, FS, MW, FP, and BS contributed to the conception and design of the study. MW organized the database. YX, SL, and HL finished the experiments. YX wrote the first draft of the manuscript. FS and LZ finalized the manuscript. All authors contributed to manuscript revision, read, and approved the submitted version.
